# Analysis of Estonian surveillance in wild boar suggests a decline in the incidence of African swine fever

**DOI:** 10.1038/s41598-019-44890-0

**Published:** 2019-06-11

**Authors:** Katja Schulz, Christoph Staubach, Sandra Blome, Arvo Viltrop, Imbi Nurmoja, Franz Josef Conraths, Carola Sauter-Louis

**Affiliations:** 1 Friedrich-Loeffler-Institut, Federal Research Institute for Animal Health, Institute of Epidemiology, Südufer 10, 17493 Greifswald, Insel Riems Germany; 2 Friedrich-Loeffler-Institut, Federal Research Institute for Animal Health, Institute of Diagnostic Virology, Südufer 10, 17493 Greifswald, Insel Riems Germany; 3 Estonian University of Life Science, Institute of Veterinary Medicine and Animal Sciences, Kreutzwaldi 62, 51014 Tartu, Estonia; 4 Estonian Veterinary and Food Laboratory (VFL), Kreutzwaldi 30, 51006 Tartu, Estonia

**Keywords:** Risk factors, Viral infection

## Abstract

African swine fever (ASF) in wild boar populations is difficult to control. In affected areas, samples from all wild boar shot and found dead are investigated. The use of laboratory tests allows estimating the duration of the infection in affected animals. The study aimed to test the hypothesis that the stage of the epidemic in different areas of Estonia can be assessed on the basis of prevalence estimates. ASF surveillance data of Estonian wild boar were used to estimate prevalences and compare them between the East and West of Estonia. The temporal trend of the estimated prevalence of ASF virus positive animals and of the estimated seroprevalence of wild boar showing antibodies against ASFV was analyzed. Due to the potential influence of population density on the course of ASF in wild boar, also population density data (number of wild boar/km^2^) were used to investigate the relationship with laboratory test results. In areas, where the epidemic had already lasted for a long time, a small number of new cases emerged recently. The prevalence of samples that were only seropositive was significantly higher in these regions as compared to areas, where the epidemic is in full progress. The observed course of the disease could be the beginning of an ASF endemicity in this region. However, the results may also indicate that ASF has started to subside in the areas that were first affected in Estonia.

## Introduction

African swine fever (ASF) is a hemorrhagic disease of suids caused by a large DNA virus of the Asfarviridae family, African swine fever virus (ASFV)^1^. The virus was introduced into Georgia in 2007. It spread from there affecting both, domestic pigs and wild boar^2^. Until now, ASF emerged also in nine countries of the European Union and in some Asian countries including China, Mongolia, Vietnam and Cambodia (OIE WAHIS interface, visited online 26^th^. April 2019).

So far, the course of the ASF epidemic in domestic pigs indicates that controlling the disease in farmed animals had been relatively successful in most, but not all countries (e.g. Romania). By contrast, eradicating ASF from an affected wild boar population appears to be difficult^3–5^. Originally, it was hypothesized that ASF in wild boar might either fade out quickly due to the high virulence of the pathogen or it will spread rapidly throughout the whole continent^4^. By now, it is obvious that none of the two scenarios became reality. Nonetheless, there is still the chance that ASF in wild boar might subside due to the increasing herd immunity, developed through the increasing proportion of surviving wild boar^6^.

It is known, that also a dense wild boar population may influence the dynamics of ASF. Most experts agree that a low population density reduces the risk of ASF spread^3,7–12^. Nurmoja, *et al*.^7^ showed a positive association between population density and the prevalence of ASF in wild boar. Due to the ongoing discussion regarding the potential role of the wild boar population density on the spread of ASF and thus its influence on the sero- and ASFV prevalences, we investigated the potential relationships between the temporal trends of prevalence estimates and the wild boar population.

Considering the known risk factors, the course of the ASF epidemic in Estonia illustrates the challenges to eliminate the disease from a wild boar population. In September 2014, ASF entered Estonia in the Southeast, probably coming from Latvia^7,13^. Further ASF cases were detected in the Northeast, 200 km away from the affected area in the South and close to the border with the Russian Federation. These cases were considered as epidemiologically independent of the cases in the South of Estonia^7,14^. The disease spread slowly, but inexorably towards the center of the country and reached the western part including the island of Saaremaa in 2016^3,7,15^. Recent surveillance data of 2018 from the whole of Estonia indicated that the number of ASF cases in sampled wild boar has decreased. Initial statistical analyses also pointed at a clear decrease of the number of ASFV-positive wild boar, especially in eastern Estonia, where the epidemic started^3^. An increase of samples that were only seropositive, but ASFV-negative, has also been noticed^3^.

We thus aimed to investigate in the present study, whether there is a difference in the surveillance data, namely the laboratory test results, between areas, where ASF emerged in 2014 (i.e. the eastern part of Estonia) and regions, where the epidemic did not start before 2016 (i.e. the western part of Estonia). Correspondingly, we tried to assess the current epidemiological situation based on laboratory test results and tested the hypothesis that a decrease of ASFV positive cases combined with a slight increase of seropositive cases, suggesting an increased herd immunity, might indicate a decline of the incidence of ASF in Estonian wild boar.

## Material and Methods

### Study area and study period

**Figure 1:**
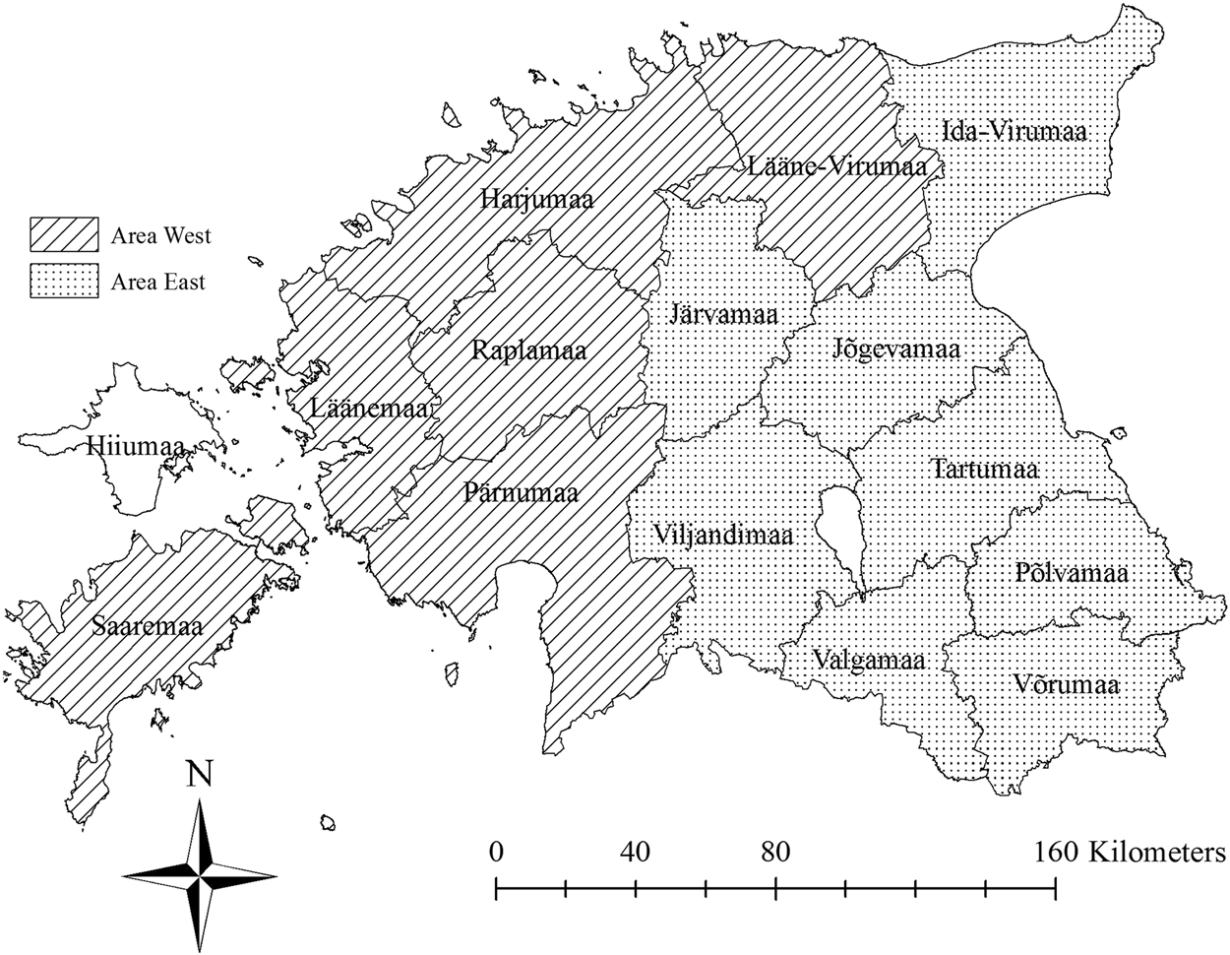
The study areas “East” and “West” in Estonia. The island of Hiiumaa was excluded. Map was generated by using ArcGIS ArcMap 10.3.1 (ESRI, Redlands, CA, USA).

Estonia was divided into two areas, “East” and “West”. Only the Island of Hiiumaa was excluded from the study area because it remained free from ASF so far. The eastern part of Estonia (area “East”) consisted of eight counties and the western part of the country (area “West”) of six counties (Fig. 1). Area “East” included the counties, where ASF newly emerged in 2014 and that were fully affected by ASF by the end of 2015 and area “West” the counties in which first sporadic cases emerged in the middle of 2015 and the extensive spread started in January 2016.

The sizes of the two study areas were calculated on the basis of hunting grounds to avoid biased results due to cities or large bodies of water. The sizes of the hunting grounds in each county were added up and the total was used for calculations. Therefore, area “East” included a size of 19,611.72 km^2^ and area “West” a size of 20,283.23 km^2^.

The study period comprised 44 months and lasted from December 2014 to July 2018.

### Data

Surveillance data were extracted from the CSF/ASF wild boar surveillance database of the European Union (https://surv-wildboar.eu). The database includes one data record for each sampled wild boar. Each record contains information about the origin of the sample (from animals found dead or hunted apparently healthy), the sampling location, age and gender of the sampled animal and the results of serological and virological laboratory tests (i.e. detection of ASFV by qPCR^16^). For the serological tests, a commercially available ELISA (Ingezim PPA COMPAC, Ingenasa, Madrid, Spain) was used. Further details about the sampling procedures and the laboratory tests have been described elsewhere^7^. Age was categorized by attributing wild boar younger than one year to one group and those older than one year into another group. Data from passive surveillance included samples originating from animals that had been found dead, were shot sick or died in a road traffic accident. All samples that yielded an inconclusive test result for ASFV were excluded from the analyses.

Spatial analyses were based on the administrative reform of municipalities in 2017. Data records from 2014–2017 were assigned to the new administrative units (eight counties for area “East” and six counties for area “West”) according to their coordinates using the software ArcGIS ArcMap 10.3.1 (ESRI, Redlands, CA, USA (http://www.esri.com/).

Wild boar population data were obtained from the Estonian Environment Agency (Nature department) and were based on estimates provided by hunters. Population density data for wild boar in Estonia have previously been described in detail^7^.

For the present study, population data estimates were available as integer numbers of wild boar. The respective information was available for six hunting years (April 2012/March 2013 to April 2017/March 2018) and for each county of the study area. The areas (in km^2^) of hunting grounds in each county were summed and the estimated wild boar density (wild boar/km^2^) in each county was determined based on the calculated size of areas that could function as wild boar habitats (e.g. water bodies and streets were excluded).

### Data analyses

The study period was divided into a first half, including data from Dec 2014–Sept 2016 (first 22 months of study period) and a second half consisting of data from Oct 2016–July 2018 (second 22 months of study period).

We analyzed the estimated ASFV prevalence and seroprevalence by grouping the samples as described in the following section.

**Figure 2:**
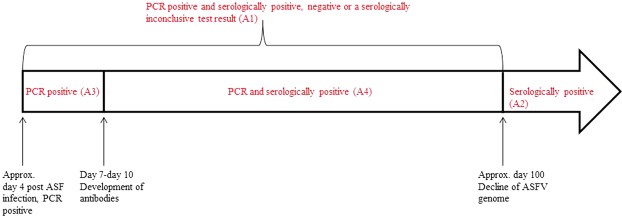
Timescale illustrating the course of laboratory test results for African swine fever.

The estimated prevalence was calculated for all samples that had yielded an ASFV- positive PCR result and either a positive, negative or inconclusive test result for specific antibodies directed against ASFV (designated as group A1, Fig. 2). Prevalences were also calculated for all samples that had tested positive for ASFV-specific antibodies and had yielded an ASFV-negative PCR result (designated as group A2, Fig. 2). We assumed that the former group (A1) represents animals in the active phase of infection and that the latter one (A2) consists of convalescent wild boar. For the sake of completeness, the estimated prevalence for samples that had tested only PCR-positive, but seronegative (group A3, Fig. 2), as well as for samples that had tested positive for ASFV and simultaneously for antibodies against ASFV (group A4, Fig. 2) were calculated separately.

The estimated prevalences based on detection of viral genome by PCR and antibody detection by ELISA (group A1 and group A2, Fig. 2) were first calculated separately for each study area (area “East” and area “West”). Within each study area, the prevalence estimates were compared between the first and the second half of the study period. Fisher’s exact test was used to test for statistical significance. A p-value of ≤0.05 was regarded as statistically significant and 95% confidence intervals were determined on the basis of Clopper and Pearson^17^. The described analyses were done by using the software package R (http://www.r-project.org)^18^.

To investigate the temporal courses of the raw prevalence estimates (groups A1 and A2, Fig. 2) a hierarchical Bayesian space–time model was used. Due to random variation in the estimated prevalence regarding the individual geographical units and time, we adjusted for spatial effects and season. The model was adapted from Staubach, *et al*.^19,20^ and analyses were performed applying BayesX 2.0.1 (http://www.uni-goettingen.de/de/bayesx/550513.html) as previously described^7,20,21^. Age and origin of the samples were defined as fixed independent variables and time, space and season were included random factors. The corresponding prevalence constituted the dependent variable. A Markov Chain Monte Carlo algorithm (MCMC) was implemented to obtain parameter estimates. 50,000 iterations were performed and at every 50^th^ iteration, a sample was selected. For the burn-in 1,000 iterations were chosen. A detailed description of the model can be found in the Supplementary Information.

Differences in the population densities (number of wild boar/km^2^) between hunting years were calculated for each study area and statistically analyzed using the non-parametric Kruskal-Wallis test. To assign statistically significant differences to the respective hunting years, a Mann–Whitney U test was used for pairwise comparisons. Bonferroni correction was applied to control for the type I-error in multiple testing scenarios^22^. Differences between the two studies areas in individual hunting years were also analyzed using the Mann–Whitney U test. The analyses regarding the population density were performed using the software package R (http://www.r-project.org)^18^.

### Maps and figures

Maps were generated using ArcGIS ArcMap 10.3.1 (ESRI, Redlands, CA, USA, http://www.esri.com/). Figures were generated using the software package R (http://www.r-project.org)^18^.

## Results

### Data

After exclusion of data sets originating from the island of Hiiumaa (N = 1,255) and all data resulting in an inconclusive virological test (N = 114), a total of 36,456 records were available for analyses. 56.95% of all samples were investigated in the first study half (December 2014 – September 2016). In the area “East”, 13,455 samples were examined, of which 10,887 had been investigated in the first half of the study period (December 2014 – September 2016). In the area “West”, 23,001 samples were obtained, 9,875 thereof in the first half of the study period (December 2014 – September 2016) (Table 1).

**Table 1:** Total number of investigated samples (by PCR for ASFV or ELISA for antibodies against ASFV), the numbers of all samples that tested positive for ASFV (irrespective of the serological test result = > group A1) or were seropositive (with a negative ASFV PCR result = > group A2), the calculated prevalence (95% confidence intervals) and the statistical significance of the difference in the prevalences between study area “East” and study area “West” in the first and the second half of the study period.

Study months	Study area	Number of samples tested for ASFV by PCR	Number of PCR- positive samples (A1)	Prevalence (95% confidence interval)	p-value	Number of samples tested for antibodies against ASFV	Number of sero positive samples (A2)	Seroprevalence (95% confidence interval)	p-value
1–22	“East”	10,887	1,497	0.138 (0.131–0.144)	<0.001	9,700	203	0.021 (0.018–0.024)	<0.001
	“West”	9,875	459	0.046 (0.042–0.051)		9,462	55	0.006 (0.004–0.008)	
23–44	“East”	2,568	52	0.020 (0.015–0.026)	<0.001	2,518	165	0.066 (0.056–0.076)	<0.001
	“West”	13,126	889	0.068 (0.063–0.072)		12,600	358	0.028 (0.026–0.031)	

### Data analyses

For the first half of the study period (December 2014 – September 2016), analysis yielded a statistically significantly higher estimated prevalence of samples of group A1 (ASFV PCR-positive and seropositive-, seronegative or inconclusive samples) in area “East” compared to area “West” (Table 1; Supplementary Figs S1 and S2). The same applied for the estimated prevalence of samples of group A2 (seropositive samples) (Table 1; Supplementary Figs S3 and S4). By contrast, in the last 22 months of the study period, the estimated prevalence of samples from group A1 was significantly higher in the area “West” than in area “East” (Table 1; Supplementary Figs S1 and S2). In area “East”, the estimated prevalence of seropositive wild boar (group A2) was significantly higher than in area “West” in both parts of the study period (Table 1). However, if individual months are considered, the seroprevalence did not show a statistically significant difference between the two study areas in most months (Supplementary Fig. S3).

Additional analysis of the prevalence of samples that were only PCR-positive and serologically negative (group A3) yielded a clear decrease of the estimated prevalence in the East over time (from 8% in month 12 and 20 and 0% in most of the study month of the second half of the study period, Supplementary Fig. S5 and Table S1). In area “East”, samples that were PCR and seropositive (group A4) showed a high prevalence in the first half of the study period, whereas a high prevalence was found in area “West” in the second half of the study period (Supplementary Fig. S6 and Table S1).

**Figure 3:**
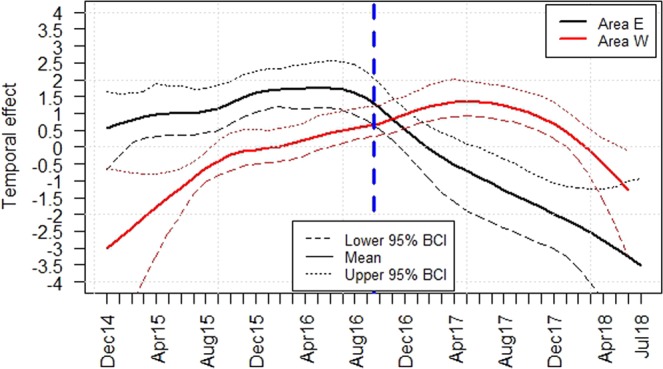
Median temporal effect of all samples from area “East” (E) and area “West” (W) that tested PCR-positive, irrespective of the serological result, on the logit prevalence. 95% Bayesian credible intervals (BCI) are indicated. Figure was generated by using the software package R (http://www.r-project.org).

**Figure 4:**
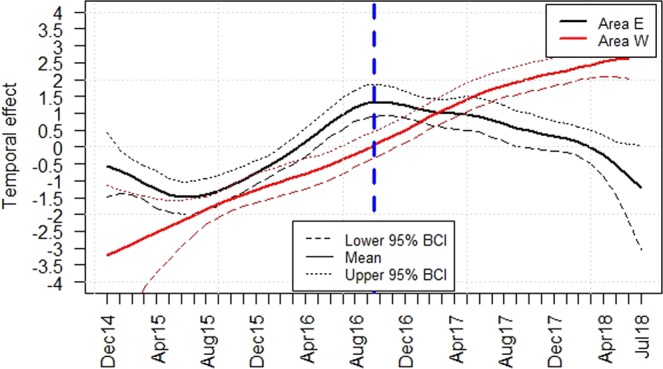
Median temporal effect of all samples from area “East” (E) and area “West” (W) that tested exclusively serologically positive on the logit prevalence. 95% Bayesian credible intervals (BCI) are indicated. Figure was generated by using the software package R (http://www.r-project.org).

After adjusting the ASFV prevalence (group A1) or the seroprevalence (group A2) for seasonal effects in the areas “East” and “West” (Supplementary Figs S7 and S8), the temporal course of the prevalence estimates was investigated (Figs 3 and 4). In area “East”, the model analyses suggested a decrease in the temporal trend of the logit prevalence of PCR-positive wild boar (group A1) in the last 22 months of the study period. Until August 2016, there was an increase of the logit prevalence of samples from group A1 in area “West”. Towards the end of the study period, the logit prevalence also appeared to decrease in area “West”. In the last four months of the study period, no significant difference in the logit prevalences between the two areas was detected (Fig. 3). In area “East”, the temporal trend of the logit prevalence showed an increase within the first half of the study period for all samples that tested positive exclusively for antibodies against ASFV (group A2). In the second half, the seroprevalence seemed to decrease slightly. By contrast, the logit prevalence of serologically positive wild boar showed a steady rise over time in area “West”. In the last year of the study period, the logit prevalence of seropositive wild boar was significantly higher in area “West” than in area “East” (Fig. 4).

**Figure 5:**
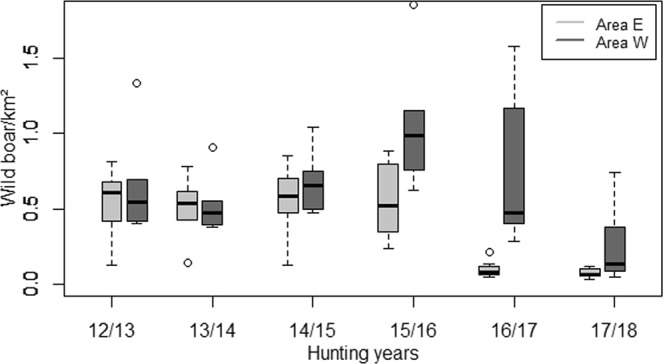
Wild boar population density (number of wild boar/km^2^) in the different hunting years for area “East” (E) and area “West” (W). The horizontal line that forms the top of the box illustrates the 75th percentile. The horizontal line that forms the bottom is the 25th percentile. The horizontal line that intersects the box is the median number of wild boar per square kilometer. Whiskers represent maximum and minimum values that are no more than 1.5 times the span of the interquartile range and the open circle represent outlier, which are single value greater or less than the extremes indicated by the whiskers. Figure was generated by using the software package R (http://www.r-project.org).

The wild boar population density (number of wild boar/km^2^) was calculated for six hunting years for area “East” and area “West“. In area “East”, the population density appeared to be stable during the hunting years 12/13–15/16 (averaged densities between 0.51 and 0.56 wild boar/km^2^). However, a significant decrease from an average of 0.56 wild boar/km^2^ to an average of 0.10 wild boar/km^2^ was found between the hunting years 15/16 and 16/17 (p = 0.001) (Fig. 5). Between 2016/17 und 2017/18, there was only a minor decrease from an average of 0.1 wild boar/km^2^ to an average of 0.07 wild boar/km^2^ (p = 0.382). In area “West”, no significant difference in the wild boar population density over the years was found (Fig. 5).

Comparing the population densities between area “East” and “West”, there was no significant difference for most of the analyzed hunting years. Only in hunting years 2015/16 and 16/17, the number of wild boar per square kilometer was significantly lower in area “East” (p = 0.02 and p < 0.001; Fig. 5).

## Discussion

The course of ASF in wild boar populations in affected countries appears to be extremely difficult to control so far. It has been hypothesized that eradicating the disease from the wild boar population in some areas may be impossible^4,7,21^. However, Mur, *et al*.^23^ suggested that ASF virus may not able to persist only in wild boar populations on the long term, although this hypothesis was made with regard to the ASF epidemic that occurred in Spain in the 1980s and 1990s. There, the wild boar density had been relatively low at that time. There is also recent evidence that ASF may have been brought under control in an affected area near Zlin in the Czech Republic (OIE WAHID interface, visited online 28. February 2019).

The outbreaks in domestic pigs were most probably the result of spill-over of ASF from wild boar to domestic pigs^24^. In Estonia, the last outbreak in a domestic pig farm was reported in September 2017. Direct or indirect (more likely) transmission from infected wild boar into domestic pig holdings seems possible, thus an infected wild boar population still constitutes a major threat to pig holdings and trade. As a consequence, complete eradication of ASF, also from the wild boar population, is desirable^24,25^.

The EU legislation (Council Directive 2002/60/EC) intends a 100% sampling of the whole hunting bag (all hunted wild boar) and all wild boar found dead in restricted areas. The hunting bag usually represents about 60–80% of the true population^26,27^. Therefore, the available sample size was higher than in many other published wild life studies^28–30^. The analyses of these comprehensive surveillance data combined with the knowledge of detection probabilities obtained from animal trials open up the possibility to understand disease dynamics and host-virus interactions in a better way^7,11,31,32^. Similar to the study of Nurmoja, *et al*.^7^, we used these data to evaluate the temporal trend of ASF within the Estonian wild boar population.

In the study, we aimed at detecting potential differences between the estimated prevalences in wild boar that may have acquired the infection recently and in animals that may be seen as true convalescents.

Firstly, we investigated the prevalence of samples that had yielded a positive PCR result regardless of the serological test result (positive, negative or inconclusive) obtained for the same sample (group A1). We assumed that these were animals that had contracted ASF within the last 100 days before they were sampled (Fig. 2). In doing so, we considered it as irrelevant for the study whether the positive PCR result indicated the presence of infectious virus (4 to 60 days pi) or just the detectability of viral genomes (up to day 100 pi). Under the assumption that ASFV PCR-positive animals have not been infected for more than 100 days, a large proportion of PCR-positive samples in a population indicate an acute epidemic with the emergence of new cases, i.e. a high incidence.

Wild boar that survive an ASFV infection for at least 7 to 10 days will develop antibodies. Subsequently, survivors will first be positive for both viral genome and antibodies for about 90 to 100 days, and then turn to antibody presence alone for an undetermined period (Fig. 2). Experimental studies could show that these animals are usually PCR-negative^6,33^. Following these findings, we secondly looked at the prevalence of samples that had been tested seropositive, but were negative for ASFV by PCR. Despite the fact that it cannot be completely excluded that some seropositive survivors showed intermittent viremia that was not detected at the time point of sampling^31^, we assume the survivors are not shedding significant amounts of virus or viral genome. The latter assumption is strengthened by recent studies that showed that survivors did not transmit the disease to naïve sentinels^6,14^. Also, the temporal course of the disease does not indicate a significant virus excretion^3^. However, it is not possible to categorically exclude the existence of carrier animals in the study area, i.e. there might be wild boar that survive the initial phase of acute ASF infection and develop a carrier state. However, if such animals exist, it will be difficult to detect them in the field and the true status of the course of the infection in individual animals remains unknown. The fact that we could detect only nine wild boar that were both ASF-positive by PCR and serology in the area “East” in the second half of the study period, may suggest that such animals are rare in this epidemiological situation. In areas, where seropositive animals dominate the ASF-positive findings, less new incident cases appear to occur, possibly indicating the late phase of the epidemic. However, it has to be emphasized that after the initial peak of serologically positive samples, ASF could still become endemic instead of fading out.

In 2014, ASF emerged in the eastern part of Estonia and spread slowly towards the West, where the broad expansion of ASF started in 2016. Based on this knowledge, the study area was divided into the areas “East” and “West”. The study period was also split into two periods for comparison. As expected, the prevalence estimates based on samples of wild boar that tested positive for ASFV by PCR and the ASFV-specific seroprevalence were significantly lower in area “West”. This was due to the later onset of disease in the western part of Estonia. The statistically significant differences in the second half of the study period supported the view that the ASF epidemic was in full progress in area “West” during the last 22 months of the study period. The higher seroprevalence in area “East” might indicate the absence of new ASF cases.

These results are also supported by the outcomes of the modelling analyses presented here (Figs 3 and 4). A hierarchical Bayesian space–time model was used to include also temporal, seasonal and spatial effects and to adjust for potential covariates such as age.

A clear temporal trend could be observed, in particular for the serological results. This tendency was also found in the study of Nurmoja, *et al*.^7^. The results of the model analyses indicate that towards the end of the study period, the seroprevalence decreased in area “East” and increased in area “West”. These findings were supported by mapping the serological prevalences (Supplementary Fig. S4). In 2018, the samples that tested positive by serology, but were negative for ASFV (group A2) seemed to increase in area “West” and decrease in the “East”. It can thus be hypothesized that the western part of Estonia has entered a new phase of the epidemic. The incidence of ASF cases decreases in this area. The decrease of seropositive samples in area “East” and simultaneously the absence of an increase of PCR positive samples at the end of the study period suggest that a reoccurrence of new ASF cases has been lacking so far.

Population density analyses have to be considered with the limitations that usually apply to population estimates in wild life even if generated with different methods. The problems and challenges associated with the use of such data have been a matter of extensive discussion^3,21,34,35^. However, there is no practical alternative to using these data as long as data that are more reliable are not available. Still, any of the results obtained with such data must be interpreted with extreme caution.

In the eastern part of Estonia, the significant decrease of population density (number of wild boar/km^2^) from the hunting years 2012/13–15/16 to the hunting years 2016/17 and 2017/18 suggests that the massive occurrence of ASF cases in the wild boar population might have led to a decrease after a while. At the same time, one can conclude that a low population density may lead to a significant reduction of the incidence of ASF cases in wild boar. This is in accord with the study of Nurmoja, *et al*.^7^ where a positive association between the population density and the incidence of ASF was demonstrated. Also in the western part of Estonia, the population density (number of wild boar/km^2^) has apparently decreased in the hunting year 2017/18. These data suggest that the epidemic had an effect on the population density and that the resulting low density may support the fading of the epidemic. However, the observed decrease in the population density could also be due to the implemented control measures (e.g. intensified hunting, targeted hunting of females and feeding ban). Regardless of the reason for the identified significant decrease, the study therefore demonstrates with real data what others have predicted^3,9,10^. An intensive reduction of the population density prior an emergence of ASF might thus be a huge advantage for controlling the disease in the case of an introduction. However, the question remains, whether it is possible to reduce the population density sufficiently, only by hunting, but perhaps also through other measures (e.g. ban of supplementary feeding, limiting wild boar reproduction, euthanasia with toxic substances, etc.).

The results of the study can raise hope that it may be possible to control ASF in a wild boar population, even in countries, where a large proportion of wild boar population is exposed. It is too early to predict whether an ASF-free status can be reached, but at least this seems no longer impossible. In any case, recording of all ASF-positive cases is of utmost importance, regardless of the type of test result, i.e. ASFV detection by PCR or serology. Yet, it is difficult to assess the temporal course of the epidemic by just looking at the case numbers. With this common practice, the official declaration that a country has gained an ASF-free status requires the complete absence of positive samples. This is hard to reach, particularly because serologically positive animals may be present for several years after virus circulation has stopped. In relation to seropositive wild boar, it should be mentioned that there is a controversial debate regarding potential carrier (i.e. hidden ASFV-positive) status of seropositive animals^6,36,37^. As long as it cannot be ruled out that such animals remain infectious, there might be the danger of a re-emergence of the disease in a previously affected region. Thus, a change of the current official requirements is unlikely, unless it can be proven, that carriers do not exist or that they are not infectious.

In studies regarding the ASF epidemic on the Iberian Peninsula, a possible eradication of ASF in wild boar was already hypothesized^23^. However, a favorable course of the disease still depends on several unknown factors which will need clarification. It is still unknown, how long protective immunity last and which level of herd immunity is sufficient for protection. Also, the role of maternal antibodies for protection against ASFV infection of young wild boar remains in contrast to classical swine fever (CSF) still unknown^38,39^. This is particularly important as wild boar have a high reproduction rate, therefore beget a huge number of potentially susceptible descendants. Furthermore, in contrast to CSF virus, which is not able to remain infectious in the environment for a longer period^38,40^, it is generally accepted that ASFV remains stable even under harsh conditions, although reliable figures about the tenacity of the virus are scarce^41,42^. Moreover, it is almost impossible to detect and to remove all dead wild boar from an affected region^43,44^. Therefore, carcasses of infected wild boar might constitute a potential source of reinfection. Probst, *et al*.^45^ found that wild boar seem to be interested in the soil around dead conspecifics and suggested therefore an efficient removal of dead wild boar carcasses. This suggestion was also supported by the European Food Safety Authority^3^. ASFV may thus still be present in area “East” despite a few months without or with a very small number of newly detected ASF cases in wild boar, and might cause a re-emergence of the disease at any time. Also, some studies suggest, that an increasing dominance of seropositive results might be the result of a rising number of surviving animals and of a potential attenuation of ASFV in these areas^14,31,37,46^. It is also known from other diseases, e.g. CSF, that the seroprevalence is usually low at the beginning of an epidemic, but that the incidence of seropositive cases increases as the epidemic proceeds, while the number of new cases decreases due to the developed natural immunity and therefore due to the decreasing number of susceptible animals. As soon as herd susceptibility returns again, the numbers of newly infected individuals may increase again^47–49^. In contrast to CSF, the currently observed seroprevalence of ASF is low. This is probably due to the high case-fatality ratio of ASF, which is much higher than that of CSF^38^. Also, in contrast to CSF, there is no vaccination against ASF, which may in the case of CSF have contributed to the increase of the seroprevalence in a CSF-vaccinated wild boar population^38,49^.

A favorable course of the disease certainly also depends on the success of implemented control measures, which need to be regularly evaluated and adapted as appropriate, However, not even the best control measures can prevent the new entry of ASF from neighboring regions. The likelihood of a new entry from neighboring countries depends certainly on the current phase of the epidemic in bordering countries, in particular in wild boar, but also on the local surveillance efforts. It is known, that there are still ASFV positive cases in the Russian Federation which is directly bordering Estonia. Finally, the unpredictable human factor, which is known to play a major role in the emergence and spread of ASF^4,13,24,50^ can always lead to the re-emergence of ASF, in particular in wild boar.

The present study may open new perspectives with regard to the evaluation of the epidemic status of ASF in wild boar. Comprehensive surveillance and laboratory results can be used to assess the status of an epidemic. Careful analyses of these data may for example help to identify areas with an increased incidence. This knowledge can then be used to adapt control measures accordingly. Follow up studies, investigating virological and environmental factors influencing e.g. immunity or ASF transmission, investigating the course of the epidemic with newly emerging surveillance data and evaluating the implemented control measures for ASF in wild boar in Estonia would be useful to bring the course of ASF in Estonia into context with the course of ASF epidemics in other countries.

In conclusion, the course of ASF in wild boar in Estonia was investigated using the available surveillance data including laboratory test results. The results of the analyses indicate a clear decline of the disease in the East of the country. This temporal course of the disease suggests that there is a chance that ASF will continue to subside in this region.

## Supplementary information

Supplementary information

## Data Availability

The original data used for the analyses can be obtained from the author after approval by the responsible institution in Estonia.
