# Supplementary material for: Analysis of Estonian surveillance in wild boar suggests a decline in the incidence of African swine fever
Source: Sci Rep. 2019 Jun 11;9:8490. doi: 10.1038/s41598-019-44890-0 (PMC6560063; doi:10.1038/s41598-019-44890-0)
Supplement: Supplementary file 1 — Supplementary information [file 41598_2019_44890_MOESM1_ESM.pdf]

Analysis of Estonian surveillance in wild boar suggest a decline in the incidence of African swine fever

Katja Schulz, Christoph Staubach, Sandra Blome, Arvo Viltrop, Imbi Nurmoja, Franz Josef Conraths, Carola Sauter-Louis

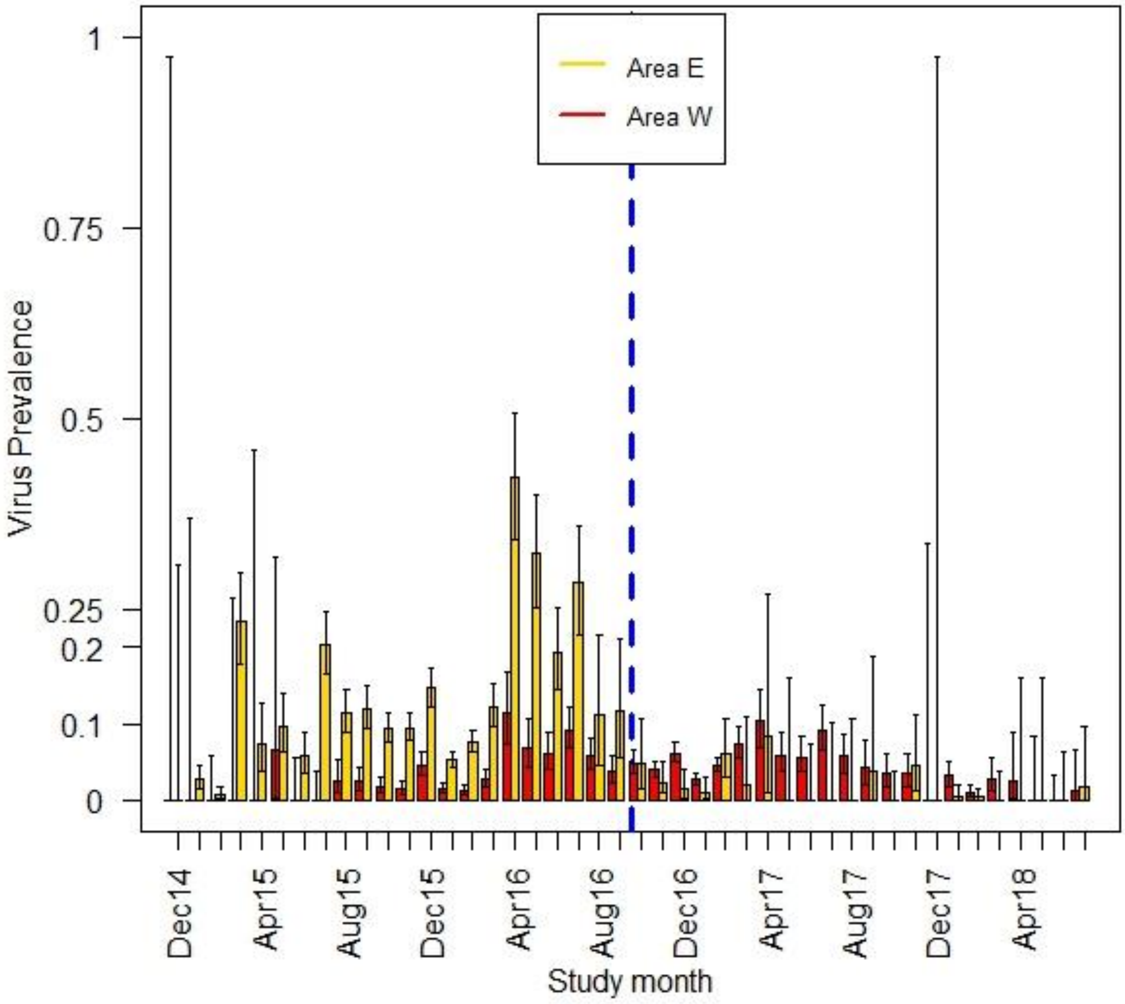

Figure S1: Prevalence estimates for ASFV PCR positive samples irrespective of their serological test result, in area "East" (E, yellow) and in area "West" (W, red) for the entire study period. The whiskers indicate 95% confidence intervals. Blue dotted line constitutes the separation between the first (month 1-22) and the second half (month 23-44) of the study period. Figure was generated by using the software package R (http://www.r-project.org).

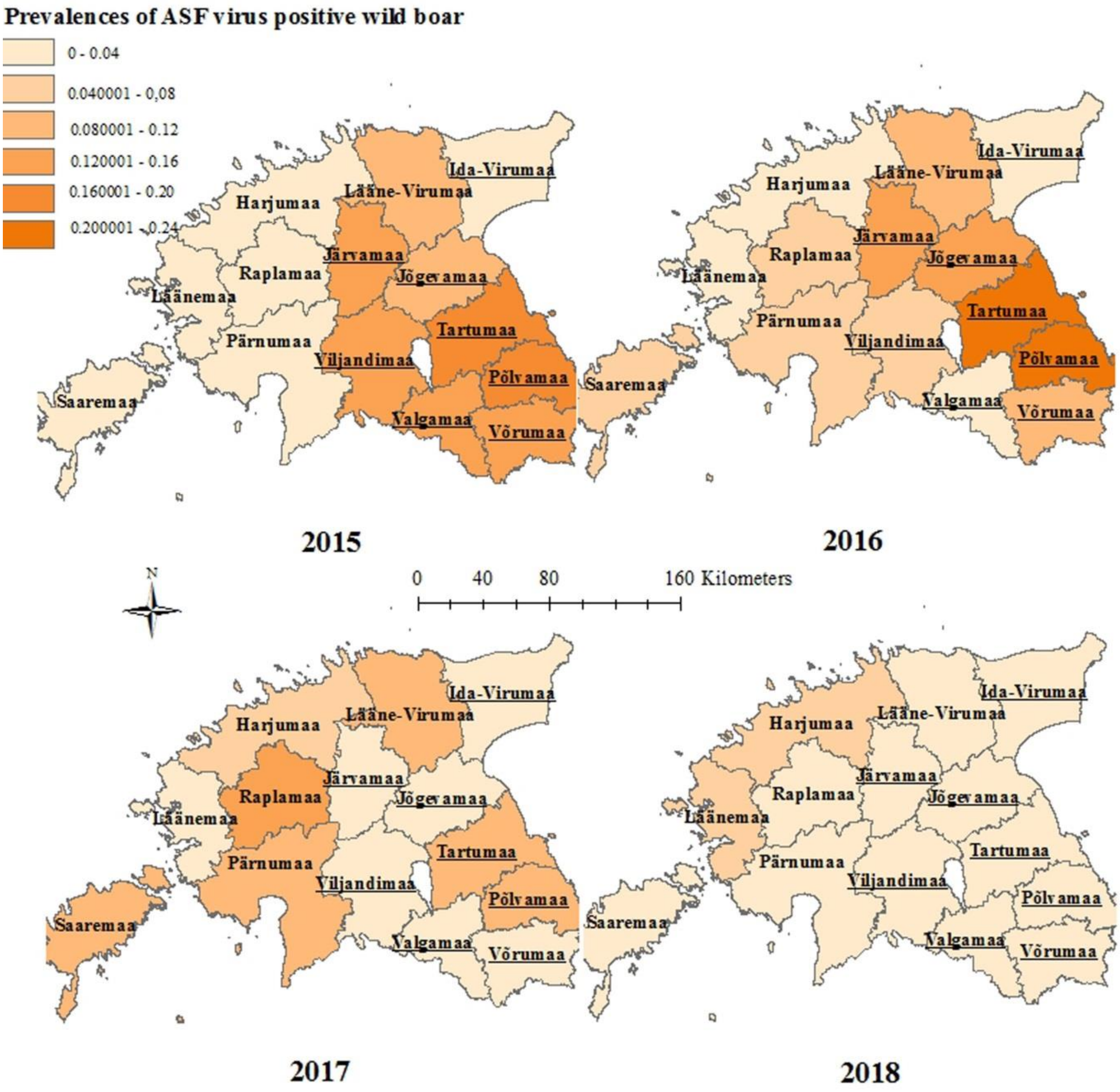

Figure S2: Prevalence estimates of all PCR positive samples irrespective of their serological test result (positive, negative or inconclusive), in the counties of area "East" (underlined counties) and in the counties of area "West" for the different years of the study period. December 2014 was excluded and the island of Hiiumaa was excluded. Map was generated by using ArcGIS ArcMap 10.3.1 (ESRI, Redlands, CA, USA).

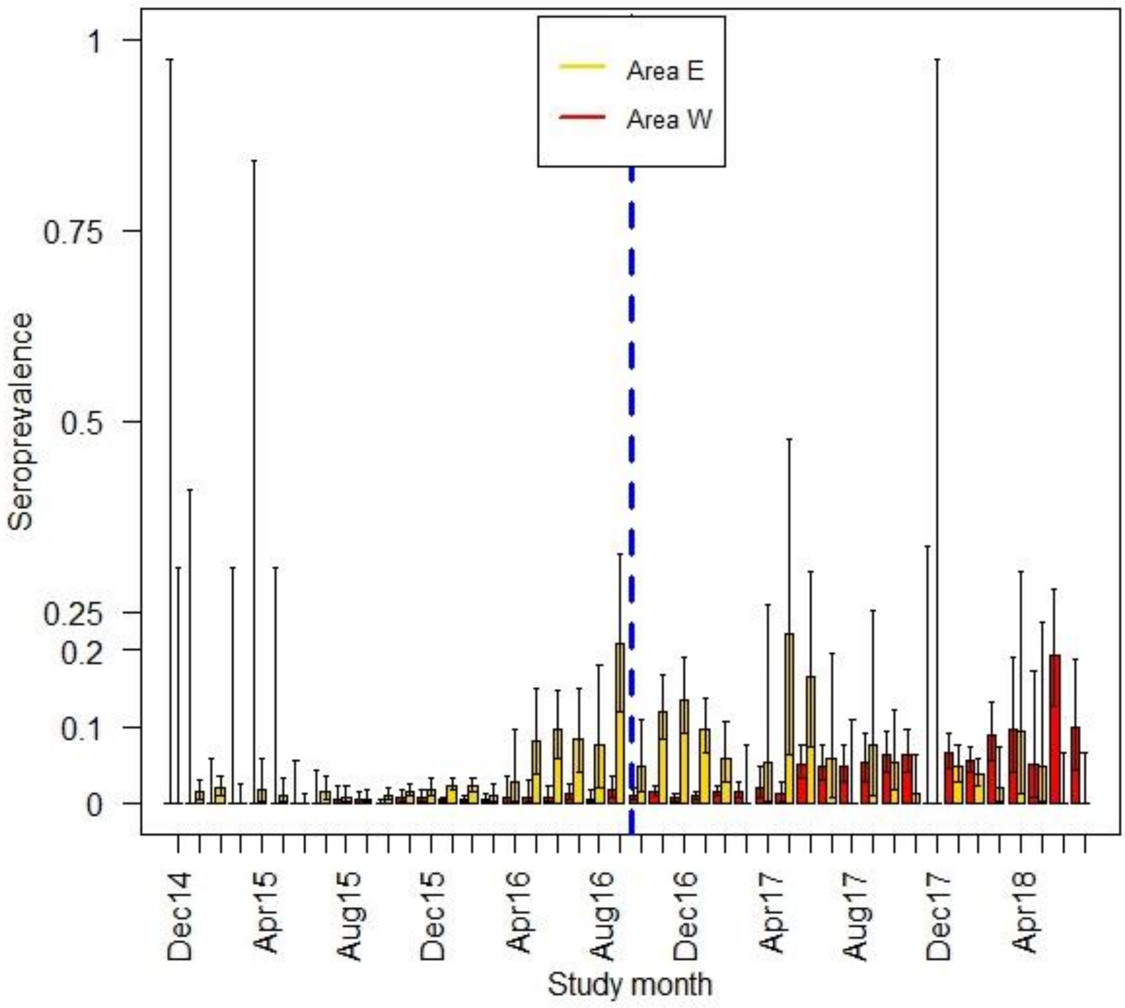

Figure S3: Prevalence estimates for ASFV antibody positive samples in area "East" (E, yellow) and in area "West" (W, red) for the entire study period. The whiskers indicate 95% confidence intervals. Blue dotted line constitutes the separation between the first (month 1-22) and the second half (month 23-44) of the study period. Figure was generated by using the software package R (http://www.r-project.org).

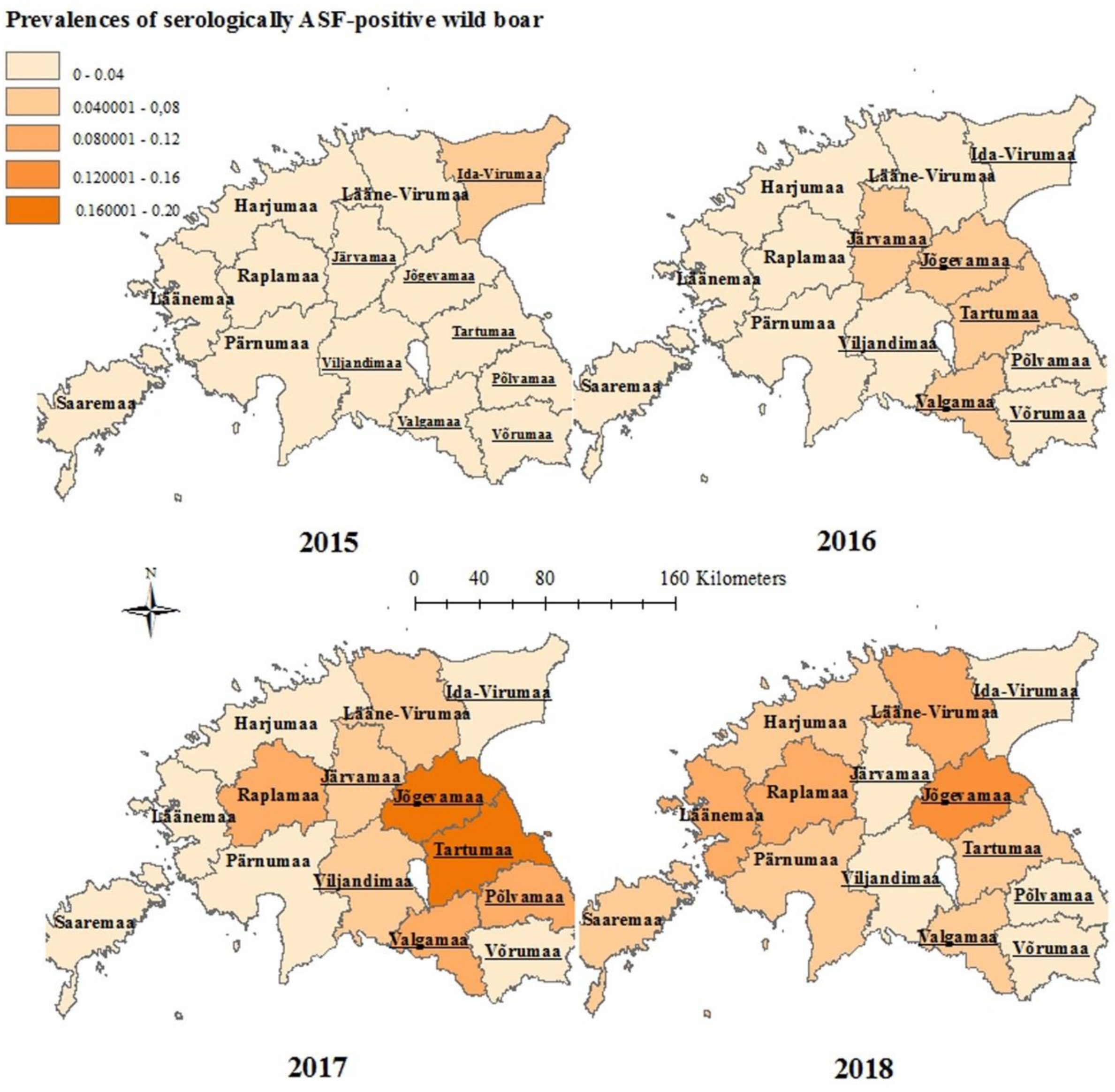

Figure S4: Prevalence estimates for serologically positive and PCR negative samples, in the counties of area “East” (underlined counties) and in the counties of area “West” for the different years of the study period. December 2014 was excluded and the island of Hiiumaa was excluded. Map was generated by using ArcGIS ArcMap 10.3.1 (ESRI, Redlands, CA, USA).

Table S1: Numbers of all samples that tested positive for ASFV and negative for ASFV antibodies (group A3) or were ASFV- and seropositive (group A4), the calculated prevalence (95% confidence intervals) and the statistical significance of the difference in the prevalences between study area "East" and study area "West" in the first and the second half of the study period.

Study months	Study area	Number of PCR- positive samples and ASFV Ab negative samples (A3)	Prevalence (95% confidence interval)	p-value	Number of PCR and sero positive samples (A4)	Seroprevalence (95% confidence interval)	p-value
1-22	"East"	371	0.038 (0.035-0.042)	< 0.001	136	0.014 (0.012-0.017)	< 0.001
	"West"	145	0.015 (0.013-0.018)		40	0.004 (0.003-0.006)	
23-44	"East"	11	0.004 (0.002-0.008)	< 0.001	9	0.004 (0.002-0.007)	< 0.001
	"West"	305	0.024 (0.022-0.027)		145	0.011 (0.010-0.014)	

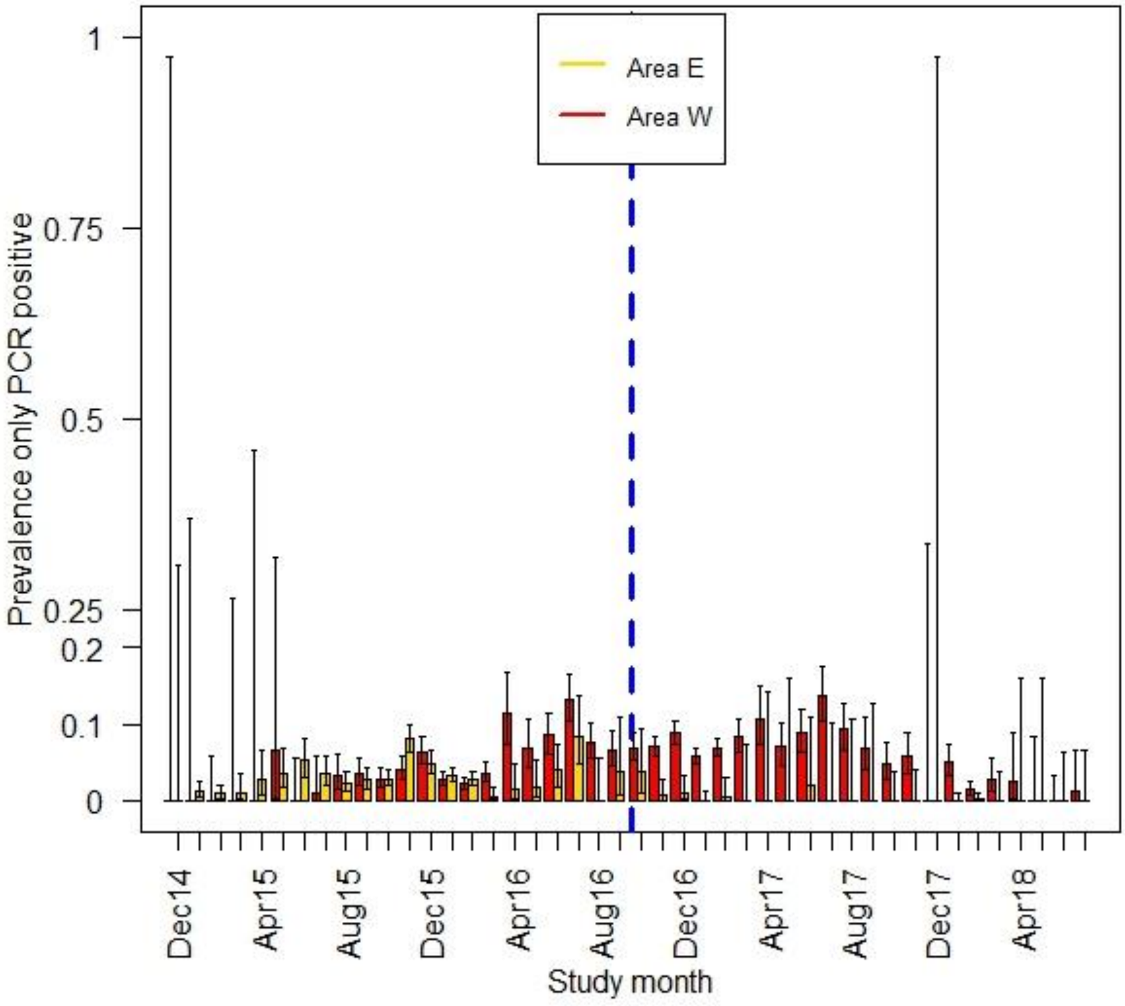

Figure S5: Prevalence estimates for ASFV PCR positive samples that were simultaneously ASFV antibody negative in area "East" (E, yellow) and in area "West" (W, red) for the entire study period. The whiskers indicate 95% confidence intervals. Blue dotted line constitutes the separation between the first (month 1-22) and the second half (month 23-44) of the study period. Figure was generated by using the software package R (http://www.r-project.org).

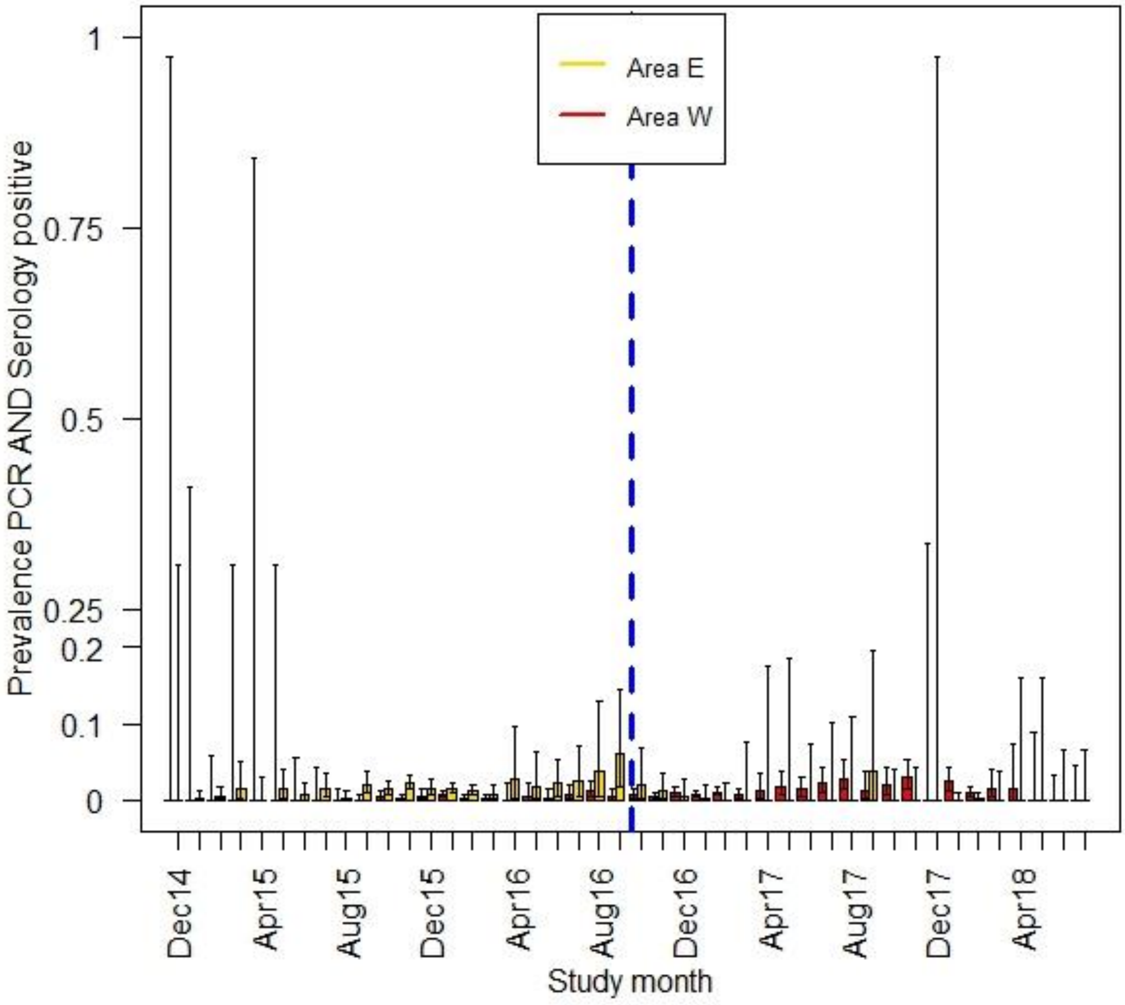

Figure S6: Prevalence estimates for ASFV PCR positive samples that were simultaneously ASFV antibody positive in area "East" (E, yellow) and in area "West" (W, red) for the entire study period. The whiskers indicate 95% confidence intervals. Blue dotted line constitutes the separation between the first (month 1-22) and the second half (month 23-44) of the study period**.** Figure was generated by using the software package R (http://www.r-project.org).

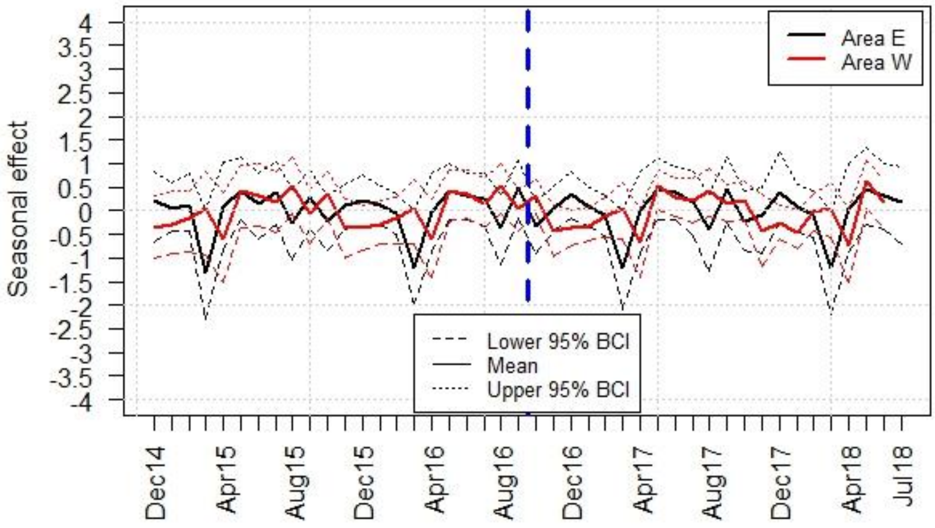

Figure S7: Median seasonal effect of all samples from area "East" (E) and area "West" (W) that tested exclusively serologically positive, on the logit prevalence. 95% Bayesian credible intervals (BCI) are indicated. Blue dotted line constitutes the separation between the first (month 1-22) and the second half (month 23-44) of the study period. Figure was generated by using the software package R (http://www.r-project.org).

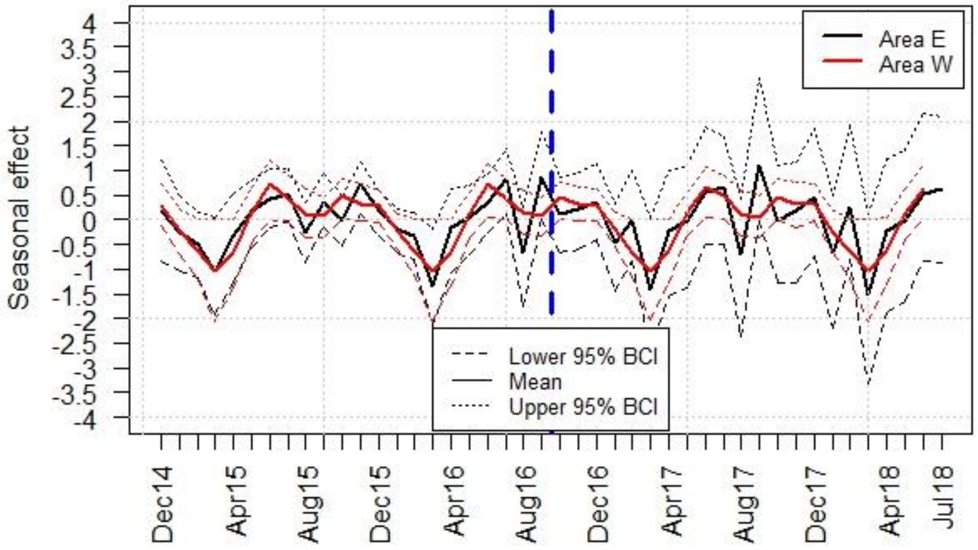

Figure S8: Median seasonal effect of all samples from area "East" (E) and area "West" (W) that tested PCR-positive, irrespective of the serological result, on the logit prevalence. 95% Bayesian credible intervals (BCI) are indicated. Blue dotted line constitutes the separation between the first (month 1-22) and the second half (month 23-44) of the study period**.** Figure was generated by using the software package R (http://www.r-project.org).

Model description (hierarchical Bayesian space–time model) adapted from Staubach, et al. [1].

The individual *i* has the unknown probability π_i that it is positive or negative for ASFV genome or ASFV antibodies depending on area *j*, in which the sampled animal lives, on the time *t*, the age (α) and the origin of sample (carcass type) (β).

The parameter π_i is modeled with a logistic model

$$\log\left(\frac{\pi_i}{1-\pi_i}\right) = \mu + \theta_j + \varphi_t + s_t + \alpha_i + \beta_i$$

μ = intercept

θ_j = spatial effect in the area *j*

φ_t = time effect on the time *t*

s_t = seasonal effect on the time *t*

α_i = fixed effect for age

β_i = fixed effect for carcass type

The structured effect θ_j displays spatial structure using a Gaussian Markov Random field (GMRF)

$$\theta_j \sim N(\theta_{neigh}/n, \sigma_\theta^2/n)$$

with $\theta_{neigh} = \sum_{k=1}^{n} \theta_k$

and *n* = no. of neighboring units *k* of unit *j*

The time and seasonal effects are modelled as a special case of Random Walk (RW) and *a priori* normal-distributed

a) RW2: time effect φ_t

$$\varphi_t \sim N(2\varphi_{t-1} - \varphi_{t-2} / \sigma_\varphi^2)$$

b) RW: seasonal effects s_t

$$s_t \sim N(-s_{t-1} - \ldots - s_{t-12-1} / \sigma_s^2)$$

For the unknown variance parameter σ^2 of the spatial effect θ, we used an inverse Gamma prior $\sigma^2 \sim$ Inv-gamma (1, 0.001). For the time effect φ and the seasonal components *s*, we chose the following inverse Gamma priors $\sigma^2 \sim$ Inv-gamma (1, 0.05).

1 Staubach, C. *et al.* Bayesian space-time analysis of Echinococcus multilocularis-infections in foxes. *Veterinary Parasitology* **179**, 77-83, doi:10.1016/j.vetpar.2011.01.065 (2011).
